# Serum procalcitonin levels associate with *Clostridioides difficile* infection in patients with inflammatory bowel disease

**DOI:** 10.1186/s12879-021-06804-2

**Published:** 2021-10-26

**Authors:** Mohammad Abdehagh, Masoumeh Azimirad, Hamidreza Houri, Banafsheh Nadalian, Fahimeh Azimirad, Meysam Olfatifar, Ome Kolsoum Nasir Shoeibi, Abbas Yadegar, Shabnam Shahrokh, Mehran Mahdavi Roshan, Hamid Asadzadeh Aghdaei, Mohammad Reza Zali

**Affiliations:** 1 Gastroenterology and Liver Diseases Research Center, Research Institute for Gastroenterology and Liver Diseases, Shahid Beheshti University of Medical Sciences, Tehran, Iran; 2 Foodborne and Waterborne Diseases Research Center, Research Institute for Gastroenterology and Liver Diseases, Shahid Beheshti University of Medical Sciences, Tehran, Iran; 3 Basic and Molecular Epidemiology of Gastrointestinal Disorders Research Center, Research Institute for Gastroenterology and Liver Diseases, Shahid Beheshti University of Medical Sciences, Tehran, Iran

**Keywords:** *Clostridioides difficile* infection, Inflammatory bowel disease, Procalcitonin level, IBD flare, Predictive biomarker, CD, ROC curve analysis

## Abstract

**Background:**

*Clostridioides difficile* infection (CDI) is a major cause of morbidity among patients with inflammatory bowel disease (IBD). Diagnostic biomarkers for early detection of CDI are needed in clinical practice. The relationship between serum procalcitonin and CDI in IBD patients has not been investigated so far. Therefore, we aimed to evaluate the usefulness of measuring serum procalcitonin level to detect CDI in patients with the flare of IBD.

**Methods:**

One hundred twenty patients with IBD were enrolled in this study. Bacterial identification was performed using standard microbiological and molecular methods. The serum procalcitonin levels were measured in all patients. Receiver operating characteristic (ROC) curve analysis was applied to assess the value of procalcitonin for the prediction of CDI among IBD patients.

**Results:**

The median serum procalcitonin level was significantly increased in IBD patients with CDI compared to non-CDI IBD patients (0.69 ng/mL vs 0.32 ng/mL). In univariate analysis, log_10_ procalcitonin was associated with CDI (OR 2.81, 95% CI 1.54–4.09, *P*-value < 0.001). Procalcitonin 1.1 ng/mL was 85% sensitive and 88% specific for the prediction of CDI. In the multivariable model including the covariates log_10_ procalcitonin, age, hospitalization, type of IBD, duration of the disease, and antibiotic usage, procalcitonin showed a robust association with CDI (OR 4.59, 95% CI 2.49–6.70, *P*-value < 0.001). An elevated procalcitonin level was associated with the presence of CDI among IBD patients.

**Conclusions:**

Our results indicate that procalcitonin level can be a good candidate biomarker for assessing the CDI in IBD patients. Further studies are required to decipher whether procalcitonin can predict CDI therapy or its recurrence.

## Background

Inflammatory bowel disease (IBD), including Crohn’s disease (CD) and ulcerative colitis (UC), is regarded as a heterogeneous chronic and relapsing inflammatory condition of the gastrointestinal (GI) tract, with an unknown etiology and unpredictable clinical course [1]. Flare-up episodes of IBD are usually characterized by abdominal pain, diarrhea, rectal bleeding, fever, and weight loss [2]. Although specific etiological factors in the development of IBD have not yet been completely elucidated, the current evidence points to a multifactorial origin, including predisposing genetic background, defects in local and systemic immune responses, the composition of the gut microbiota, and external environment [3, 4].

Although the occurrence of disease flares in IBD can be due to disease aggravation itself, it has been established that a bacterial infection with pathogenic organisms has a critical role in the deterioration and exacerbation of IBD [5]. Moreover, discriminating between disease flares and enteric infections in IBD patients is often problematic due to the similar clinical manifestations and laboratory findings [6]. Therefore, accurate early detection of enteric infections among IBD patients can prevent infection-related flares and severe complications such as bacteremia and sepsis. Importantly, the early detection of infections in IBD patients requiring immunosuppression, in which the infectious complications are facilitated in this population, has significant impact on the disease management and prognosis [7, 8]. There is clear evidence that concurrent infections with opportunistic intestinal pathogens are increasingly recognized due to immunosuppressive therapy in IBD patients [9, 10]. Apparently, patients with IBD appear to be at higher risk of severe *Clostridioides difficile* infection (CDI) than healthy people [11, 12].

The erythrocyte sedimentation rate (ESR), white blood cells (WBCs) count and C-reactive protein (CRP) level could be changed during enteric infections in IBD patients; however, these blood indexes may be elevated not only in infectious conditions but also during IBD flare-up [13, 14]. Recently, procalcitonin has been suggested as a novel biomarker for microbial infection in IBD. Procalcitonin is the precursor of calcitonin, an essential hormone produced in response to high levels of calcium in the bloodstream and involved in calcium homeostasis [15]. Serum procalcitonin is an excellent predictive factor for differentiating acute bacterial inflammatory conditions, however, it is not elevated by the inflammatory conditions of IBD itself. Literature review indicates that elevated procalcitonin levels could reflect the presence of many infective complications in IBD, including bacterial gastroenteritis and enterocolitis, postsurgical infection, intraabdominal abscess, and sepsis [16]. Furthermore, contrasted to CRP, the level of procalcitonin has been described to be unaffected during immunosuppression therapy in patients with IBD [17]. Hence, all these could be potentially taking advantage of procalcitonin to be applied as a biomarker for detection of bacterial infections for patients with IBD on  immunosuppressive treatment. However, there are some unanswered questions in the efficacy of procalcitonin and CRP measurement in IBD flare patients with concomitant CDI. This study, therefore, was conducted to explore the efficacy of serum procalcitonin measurement to detect CDI among patients with the flare of IBD.

## Materials and methods

### Patients and sample collection

Blood samples, colonic biopsies, and stool specimens were collected from 120 patients with IBD who were referred for colonoscopy at Research Institute for Gastroenterology and Liver Diseases in Tehran. Diagnosis of IBD was considered based on clinical, radiological, endoscopic, and pathological criteria, as described previously [18]. Patients were submitted to general interviews on the day of admission. A questionnaire, containing demographic data, medication, and clinical features was filled for all the patients. In our study, full remission was defined based on clinical remission and biochemical markers within normal limits. Accordingly, clinical remission was defined as a Crohn’s disease activity index (CDAI) ≤ 150 for CD patients and Mayo Clinic score ≤ 2 for UC patients, respectively [19, 20]. Biochemical remission in our evaluation was defined as a normal CRP (≤ 5 mg/L) and fecal calprotectin (≤ 150 μg/g stool) [21, 22]. In this study, a CDI case is defined as a case of diarrhea (i.e., unformed stool that conforms to the shape of a specimen collection container) that the stool sample yields a positive culture result for a toxin-producing *C. difficile* organism without other known etiologies [23]. Subjects who had received > 48 h of antibiotics or were clinically indicated to be infected with other enteric bacterial and/or viral pathogens, such as *Salmonella*, *Shigella*, *Campylobacter*, *Aeromonas*, pathogenic *Vibrio* species and *Escherichia coli* strains, and active cytomegalovirus (CMV) infection were excluded. This study was approved by the Institutional Ethical Review Committee of Research Institute for Gastroenterology and Liver Diseases at Shahid Beheshti University of Medical Sciences (Project No. IR.SBMU.RIGLD.REC.1398.041). All experiments were performed in accordance with the relevant guidelines and regulations recommended by the Research Institute for Gastroenterology and Liver Diseases. Informed consent was obtained from all subjects and/or their legal guardians before sample collection.

### Bacterial culture and isolation

For isolation of *C. difficile*, approximately 1 g of freshly stool specimens was  treated with an equal volume of ethanol, vortexed, and held at room temperature for 2 min. Homogenized stool specimens were cultured on cycloserine-cefoxitin-fructose agar (CCFA) (Mast Group Ltd., Merseyside, UK) supplemented with 7% sheep blood. In addition, colonic biopsies were transported to the laboratory in thioglycolate broth (Merck, Darmstadt, Germany) and homogenized with a tissue grinder. For identification of *C. difficile*, a hundred microliter of each homogenized specimen was cultured on CCFA. The cultured plates were incubated under anaerobic conditions (85% N_2_, 10% CO_2,_ and 5% H_2_) generated using Anoxomat^®^ Gas Exchange System (Mart Microbiology BV, Lichtenvoorde, Netherlands) at 37 °C for 48–72 h. Identification of suspected colonies was performed by standard biochemical tests. Additionally, molecular detection of *C. difficile* was carried out using amplification of the 16S rRNA gene, as previously described [24]. Stool and blood samples were also examined to rule out other infective etiology for diarrhea symptoms. Accordingly, all stool samples were examined for *Campylobacter* spp., *Shigella* spp., *Salmonella* spp. *Yersinia enterocolitica*, and pathogenic *E. coli* strains by culture method and molecular assays, as previously described [25, 26]. Moreover, blood samples of the patients were tested for anti- CMV antibodies of IgG and IgM subclasses using a commercially available ELISA kit according to the manufacturer's instructions (Vircell, Spain).

### Molecular assay for clostridial toxins

DNA was extracted from the colonies grown on specific media using DNeasy Blood & Tissue Kit (Qiagen, Hilden, Germany) according to the manufacturer’s procedure. The quality and purity of the extracted DNA were measured using NanoDrop (Thermo Scientific, Asheville, NC, USA). The presence of toxin genes of *C. difficile* was examined using PCR by specific primers, as previously described [27, 28].

### Procalcitonin and CRP measurement

The serum procalcitonin level was measured in patients with IBD using VIDAS^®^ B·R·A·H·M·S PCT™ Assay (bioMérieux Inc., Durham, USA) according to the manufacturer’s instructions. The normal range of serum procalcitonin levels was 0–0.5 ng/mL. The serum CRP levels were determined by using commercially available assay kits (Roche Diagnostics GmbH, Mannheim, Germany). The normal serum CRP level was considered to be < 0.8 mg/dL.

### Statistical analysis

Data were presented as median (interquartile range [IQR]) or n (%). Comparisons between groups (CDI + and CDI− groups) were performed by Mann–Whitney or T-test for continuous variables according to data distribution. For the diagnostic evaluation of procalcitonin, the sensitivity and specificity for the calculated cut-off point were recorded. Receiver operating characteristic (ROC) curve analysis was conducted to determine the diagnostic performance of procalcitonin in detecting CDI among IBD patients. Accordingly, we estimated the area under the curve (AUC) for procalcitonin assay compared to the culture of *C. difficile* in four fitted models, separately. In model 1, we used univariate logistic regression to test if log10 procalcitonin was significantly associated with a CDI +. In Model 2, we used multiple logistic regression with the following additional risk factors to see if they were associated with a CDI + : age, hospitalization, type of IBD (CD or UC), duration of the disease, frequency of defecation per day, and antibiotic usage. In Model 3, we reconstructed a multiple logistic regression with CRP and ESR to examine how these biomarkers interacted with procalcitonin. The final multivariable model was fitted for log10 procalcitonin based on the phase of IBD (i.e., flare or remission) (Model 4). The diagnostic performance of our models was assessed by R software version 3.6, package ROCR [29]. Other statistical analyses were performed using SPSS software version 22.0 (Chicago, IL, USA). A *P*-value < 0.05 was considered to indicate statistical significance.

## Results

### Baseline demographics and clinical characteristics

One hundred twenty IBD patients (mean 36.08 ± 14.37 years; 67 females and 53 males) consisting of 111 (92.5%) UC and 9 (7.5%) CD were included in the study. The demographic and clinical characteristics of the patients are summarized in Table 1. IBD remission was observed in 23.4% (26/111) of UC and 44.4% (4/9) of CD patients, while flare phase was found in 76.6% of UC (85/111) and 55.5% of CD (5/9) patients. The extent of disease involvement in UC patients included pancolitis 9% (10/111), extensive colitis 3.6% (4/111), left-sided colitis 10.8% (12/111), proctitis 60.4% (67/111), backwash ileitis 16.2% (18/111), Crohn colitis 55.5% (5/9) and Crohn ileitis 44.4% (4/9).

**Table 1:** Demographic data and clinical characteristics of the IBD patients

Characteristics	UC (*n* = 111)		CD (*n* = 9)	
	Flare (*n* = 85)	Remission (*n* = 26)	Flare (*n* = 5)	Remission (*n* = 4)
Gender				
Female	50 (58.8)	14 (53.8)	3 (60)	0
Male	35 (41.2)	12 (46.2)	2 (40)	4 (100)
Age				
1–20	4 (4.7)	5 (19.2)	0	0
21–30	28 (32.9)	11 (42.3)	1 (20)	3 (75)
31–40	21 (24.7)	3 (11.5)	1 (20)	0
41–50	18 (21.2)	2 (7.7)	2 (40)	0
51–60	5 (5.9)	4 (15.4)	0	1 (25)
61–70	5 (5.9)	1 (3.9)	1 (20)	0
71–80	4 (4.7)	0	0	0
Extent of disease				
Pancolitis	7 (8.2)	3 (11.5)	0	0
Extensive colitis	4 (4.7)	0	0	0
Left-sided colitis	10 (11.8)	2 (7.7)	0	0
Proctitis	55 (64.7)	12 (46.2)	0	0
Backwash ileitis	13 (15.3)	5 (19.2)	0	0
Crohn colitis	0	0	1 (20)	4 (100)
Crohn ileitis	0	0	4 (80)	0
Consistency of stool				
Watery	51 (60)	6 (23.1)	4 (80)	2 (50)
Loosed	34 (40)	20 (76.9)	1 (20)	2 (50)
Bloody	39 (45.9)	0	2 (40)	1 (25)
Clinical manifestation				
Anorexia	14 (16.5)	3 (11.5)	0	1 (25)
Abdominal tenderness	22 (25.9)	4 (15.4)	3 (60)	2 (50)
Nausea and vomiting	23 (27.1)	9 (34.6)	3 (60)	2 (50)

*IBD* inflammatory bowel disease, *UC* Ulcerative colitis, *CD* Crohn’s disease

### Microbial assessment

Totally, 17 (14.2%) patients with IBD were CDI positive, in which 34 *C. difficile* isolates were recovered simultaneously from stool and biopsy samples of these patients. *C. difficile* was isolated from 15.3% (13/85) of UC patients with flare and 11.5% (3/26) in remission. Only one isolate (20%) of *C. difficile* was isolated from a CD patient with the flare of the disease. All *C. difficile* isolates harbored genes encoding toxins A and B.

### Serum procalcitonin, CRP, and ESR levels in CDI and non-CDI groups

**Fig. 1:**
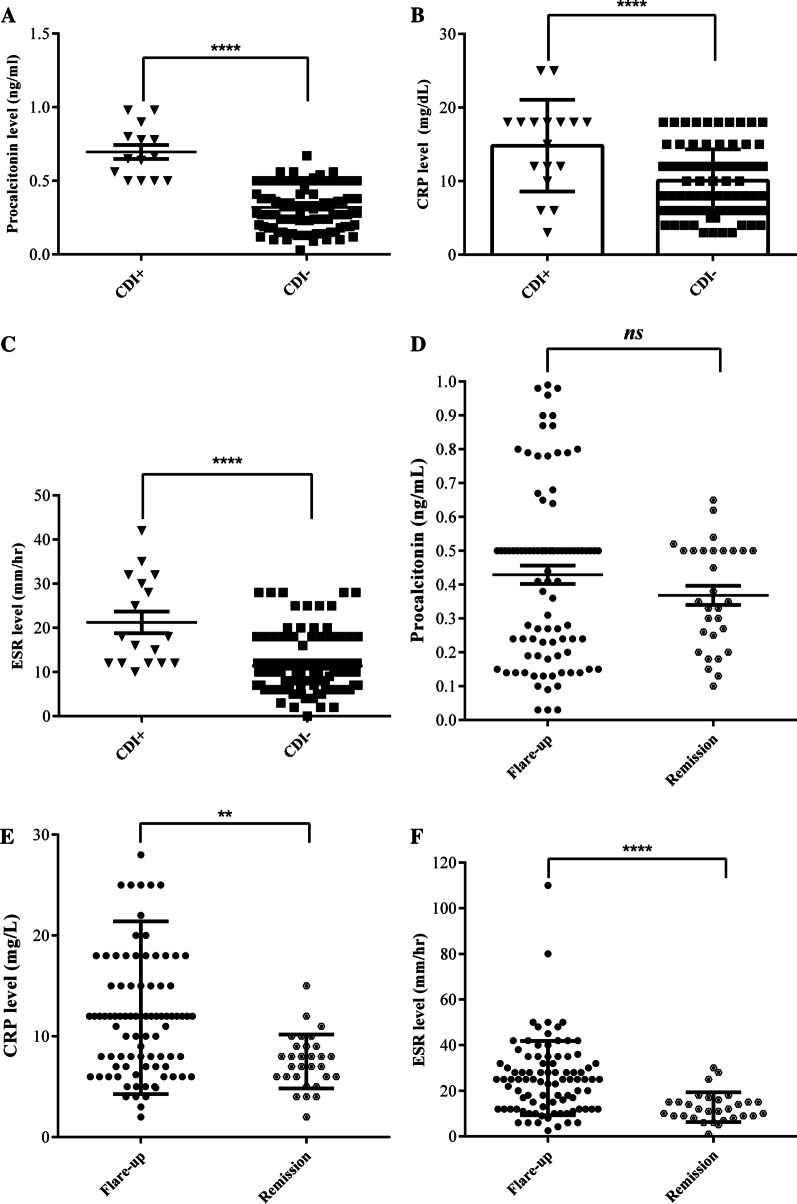
Scattergrams of serum procalcitonin levels (**A**), CRP (**B**), and ESR (**C**) in IBD patients with CDI (*n* = 17) and IBD patients without CDI (*n* = 103). Graphs (**D**–**F**) represent the levels of procalcitonin, CRP, and ESR in IBD patients with flare (*n* = 30) compared to IBD patients in remission (*n* = 90), respectively. Error bars represent the median interquartile range (IQR). The student’s t-test was used for data analysis. **, **** and *ns* denote *P*-value < 0.01, *P*-value < 0.0001 and non-significant, respectively

Serum PCT, CRP, and ESR levels concerning to CDI or non-CDI, as well as flare or remission are shown in Fig. 1. The median (IQR) serum procalcitonin levels in CDI group and non-CDI group were 0.69 ng/mL (0.5–0.98 ng/mL) and 0.32 ng/mL (0.03–0.67 ng/mL), respectively. Serum procalcitonin level was significantly increased in IBD patients with CDI compared to non-CDI IBD patients (*P*-value < 0.0001), however, this difference was not observed between IBD patients with and without a flare (Fig. 1A vs D). The median (IQR) serum CRP level in IBD patients with CDI was also higher than that for patients without CDI, 14.82 mg/dL (13.2–16.3 mg/dL) vs 10.06 mg/dL (9.4–10.5 mg/dL), respectively, (*P*-value < 0.001). Moreover, ESR level in patients with CDI displayed significantly increased (21.24 mm/h [18.12–23.83 mm/h]) compared to non-CDI group (11.41 mm/h [10.77–11.64 mm/h]) (*P-*value < 0.0001).

### ROC curve, sensitivity, and specificity

**Fig. 2:**
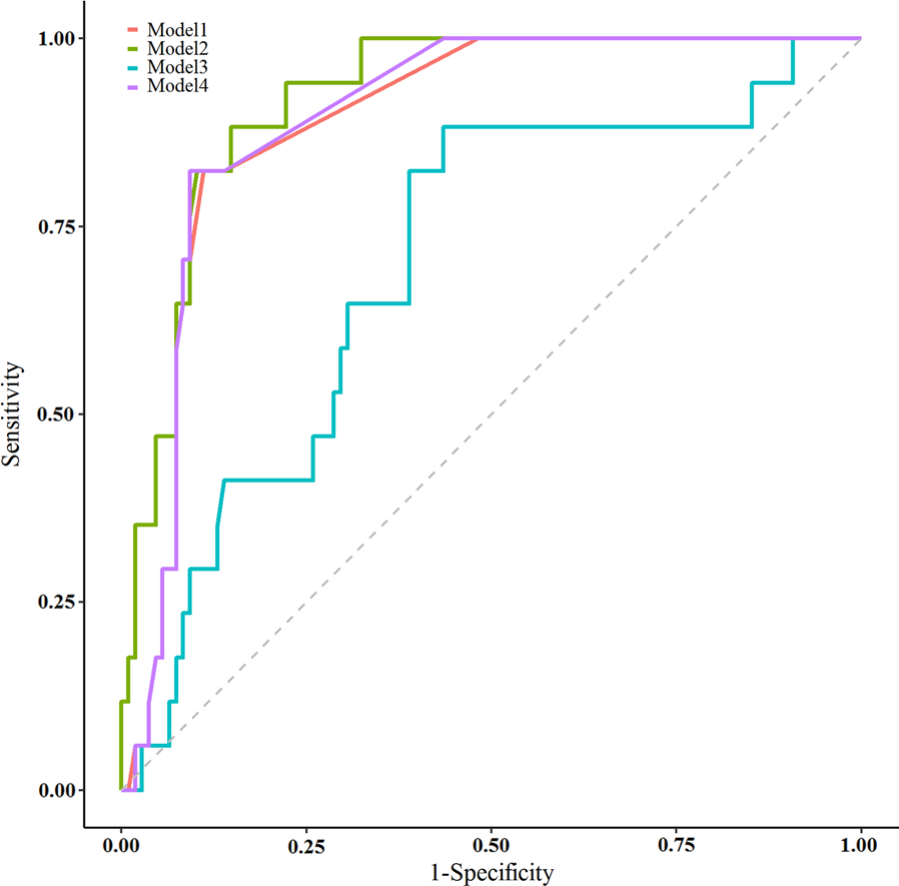
Receiver operating characteristic (ROC) curves for procalcitonin were reconstructed in 4 models. In Model 1, the estimated area under the curve (AUC) for procalcitonin was calculated at 0.89 (95% CI 0.82–0.95) by univariate logistic regression. The AUC of Model 2 was increased to 0.92 (95% CI 0.86–0.97), the sensitivity was 88% and the specificity was 88%. In Model 3, the AUC was decreased to 0.7 (95% CI 0.56–0.83), which was associated with a sensitivity of 56% and specificity of 88%. Finally, Model 4 revealed that the AUC was 0.89 (95% CI 0.83–0.95) by the sensitivity of 85% and specificity of 90%

By univariate logistic regression, log_10_ procalcitonin was associated with CDI (OR 2.81, 95% CI 1.54–4.09, *P*-value < 0.001) (Fig. 2). In the ROC curve analyses, the AUC for procalcitonin was 0.89 (95% CI 0.82–0.95). The best cut-off value for procalcitonin to diagnose CDI was 1.1 ng/mL; this threshold value was associated with sensitivity and specificity of 85% and 88%, respectively. In Model 2, which included log_10_ procalcitonin, age, hospitalization, type of IBD (CD or UC), duration of the disease, frequency of defecation per day, and antibiotic usage, procalcitonin showed a robust association with CDI (OR 4.59, 95% CI 2.49–6.70, *P*-value < 0.001) (Fig. 2, Table 2). The AUC of Model 2 was increased to 0.92 (95% CI 0.86–0.97), the sensitivity was 88% and the specificity was 88%. In Model 3, which included log_10_ procalcitonin, CRP, and ESR, procalcitonin continued to show a strong association with CDI (OR 2.85, 95% CI 1.55–4.16, *P*-value < 0.001) (Fig. 2, Table 2). However, the AUC was decreased to 0.7 (95% CI 0.56–0.83), which was associated with a sensitivity of 56% and specificity of 88%. Furthermore, log_10_ procalcitonin remained a significant predictor of CDI after adjusting for the flare phase of the disease, Model 4, (OR 2.81, 95% CI 1.53–4.09, *P*-value < 0.001) (Fig. 2, Table 2). Accordingly, our analysis revealed that the AUC of adjusted procalcitonin and flare phase of the disease in predicting CDI was 0.89 (95% CI 0.83–0.95) by the sensitivity of 85% and specificity of 90%.

**Table 2:** Odds ratios for predictors of CDI in IBD patients based on univariate (Model 1) and multiple (Models 2–4) logistic regression

Variables	Odds ratio	[95% Conf. Interval]	*P*-value
Model 1			
Procalcitonin	2.81	1.54–4.09	** < 0.001**
Model 2			
Procalcitonin	4.59	2.49–6.70	** < 0.001**
Age	1.01	0.97–1.06	0.45
UC	0.11	0.01–0.97	**0.04**
CD	9.09	2.01–53.97	** < 0.001**
Hospitalization	0.20	0.00–5.04	0.33
IBD duration	0.91	0.79–1.05	0.21
Antibiotic usage	6.87	1.12–41.98	**0.03**
Model 3			
Procalcitonin	2.85	1.55–4.16	**< 0.001**
CRP	0.93	0.80–1.09	0.41
ESR	0.98	0.98–1.09	0.11
Model 4			
Procalcitonin	2.81	1.53–4.09	**< 0.001**
Flare	5.08	0.00–0.45	**0.007**

Bold values denote a statistically significant result at the *P*-value < 0.05

*CDI* *Clostridioides difficile* infection, *IBD* inflammatory bowel disease, *UC* Ulcerative colitis, *CD* Crohn’s disease, *CRP* C-reactive protein, *ESR* Erythrocyte sedimentation rate level

## Discussion

Clinical presentation and laboratory data between disease flare and CDI are often similar among patients with IBD. It is clinically challenging to early differentiate CDI from disease flare because treatment strategies according to the disease course are completely different. Importantly, in the case of CDI, early detection and appropriate therapy can lead to a good prognosis in patients with IBD [30, 31]. Moreover, the culture of *C. difficile* is the most sensitive test but is labor-intensive and results may be delayed up to 4–7 days. Although Rao and colleagues previously described that an elevated procalcitonin level is associated with severe CDI and CDI is unlikely to be severe with a serum procalcitonin level below 0.2 ng/mL [32], there is limited data available for the diagnostic power of procalcitonin to detect CDI in patients with IBD. Several studies have previously proposed that serum procalcitonin could be a useful biomarker for early diagnosis of sepsis [33], respiratory infections [34], postoperative infections [35], ventilator-associated pneumonia [36], and severe CDI [32]. To the best of our knowledge, this is the first study conducted to evaluate the diagnostic value of procalcitonin in early diagnosis of CDI in IBD patients. Compared with culture and based on the best cut-off value (1.1 ng/mL) calculated in our study, serum procalcitonin had an acceptable specificity (85%) and sensitivity (88%) in the diagnosis of CDI.

Our data showed that serum procalcitonin, CRP, and ESR levels were significantly increased among IBD patients with CDI compared to those without CDI. All these biomarkers are measured as part of routine GI investigations, particularly for UC [37], however, some authors reported that CRP and ESR are limited by their inability to distinguish enteric infections from other inflammatory conditions [38–40]. Serum procalcitonin has been reported to offer better sensitivity and specificity than CRP and ESR for the exact diagnosis of bacterial infections [41, 42]. Our data showed that there was no significant association between serum procalcitonin level and flare of IBD regardless of CDI and serum procalcitonin was only increased in flares in patients with CDI. In the line of these findings, Chung and colleagues have described that serum procalcitonin level is not affected by IBD activity itself, although they may be affected by concomitant enteric infections and is more useful than CRP to distinguish infection stage from a flare-up of the disease [43].

Though we hypothesized that serum procalcitonin level would associate with other factors such as prolonged antibiotic courses, stage of disease, IBD duration, and type of IBD (CD or UC). We conducted multivariate analyses adjusted with procalcitonin to determine the exact relationship between this biomarker and CDI. Importantly, determination of previous antibiotic usage, type of IBD, and stage of the disease could be adjusted to maximize the prediction of CDI in patients with IBD. Although there existed a strong association between procalcitonin measurement and CDI diagnosis in the adjusted model with CRP and ESR, the sensitivity of the test was dramatically decreased. Additionally, according to our analysis (Model 4), procalcitonin measurement in IBD patients during the flare-up phase more robustly predicts CDI, thus, it may be applied for diagnosis and management of CDI in this group of patients.

This study was limited by enrolling a relatively small number of IBD patients with underlying CDI. In particular, the number of IBD patients with concomitant CDI was not equal to IBD patients without CDI. Second, the subjects were enrolled retrospectively from a single tertiary center, which may have caused an unavoidable selection bias. Furthermore, we could not follow up the subjects to evaluate the procalcitonin level before and after antibiotic treatments to know the relationship between the change in procalcitonin levels and treatment of CDI. Finally, we did not evaluate co-infection with other enteric bacterial or viral agents to whether procalcitonin could discriminate between CDI and other enteric co-infections.

## Conclusion

Our study indicated that the measurement of serum procalcitonin level can be useful in the early detection of CDI complications in IBD patients. A higher serum procalcitonin level may reflect the CDI in the stage of the flare-up. Therefore, serum procalcitonin level can be a candidate biomarker for assessing the CDI in IBD patients. However, future large-scale investigations should be performed to validate our results, to extend them to understand whether procalcitonin level measured at the flare-up stage predict CDI, and to define the role of this biomarker in treatment algorithms for CDI.

## Data Availability

The datasets used and/or analyzed during the current study available from the corresponding author on reasonable request.

## Abbreviations

CDI: *Clostridioides difficile* Infection
*Clostridioides difficile*: *C. difficile*
IBD: Inflammatory bowel disease
ROC: Receiver operating characteristic
CD: Crohn’s disease
UC: Ulcerative colitis
GI: Gastrointestinal
ESR: Erythrocyte sedimentation rate
WBCs: White blood cells
CRP: C-reactive protein
CDAI: Crohn’s disease activity index
CCFA: Cycloserine-cefoxitin-fructose agar
